# Leptomeningeal Carcinomatosis and Palliative Care: A Case Report

**DOI:** 10.7759/cureus.35615

**Published:** 2023-02-28

**Authors:** Nandan M Shanbhag, Mehad M Elbakheit, Muhammad Z Javed, Abdulghani Elomami, Afroz Samad, Bahaa E Abdulrahman, Huzaifa G Ibrahim

**Affiliations:** 1 Department of Oncology/Palliative Care, Tawam Hospital, Al Ain, ARE; 2 Department of Oncology/Radiation Oncology, Tawam Hospital, Al Ain, ARE; 3 Department of Nursing, Tawam Hospital, Al Ain, ARE; 4 Department of Pathology, Tawam Hospital, Al Ain, ARE

**Keywords:** pain management, symptom control, patient wish, palliative care, leptomeningeal carcinomatosis (lc)

## Abstract

Leptomeningeal carcinomatosis (LC) is a rare but serious complication of cancer in which cancer cells spread to the leptomeninges, the membranes that surround the brain and spinal cord. The diagnosis and treatment of LC can be challenging due to the non-specific symptoms and the difficulty of accessing the leptomeninges for a biopsy. In this case report, we describe a patient with advanced breast cancer who was diagnosed with LC and underwent treatment with chemotherapy. Despite aggressive treatment, the patient's condition worsened over time, and she was referred to palliative care, where adequate symptom control was achieved, and she was discharged to her home country as per her wish. Our case highlights the difficulties associated with the diagnosis and treatment of LC and the need for continued research to improve outcomes for patients with this condition. It specifically highlights the approach a palliative care team can take for this condition.

## Introduction

Leptomeningeal carcinomatosis (LC) is a rare but serious complication of cancer that occurs when cancer cells spread to the leptomeninges, which are the membranes that surround the brain and spinal cord [1]. LC is a devastating condition that can cause neurological symptoms and is difficult to diagnose and treat effectively.

The leptomeninges is composed of two layers: the pia mater, which is closest to the surface of the brain and spinal cord, and the arachnoid mater, which is a more fibrous layer that covers the surface of the brain and spinal cord. The cerebrospinal fluid (CSF) that surrounds the brain and spinal cord is contained within the leptomeninges [2].

The most common type of cancer that spreads to the leptomeninges is breast cancer, although it can also occur in other types of cancer such as lung cancer, melanoma, and other types of solid tumors [3]. LC is often a late complication of cancer and is typically seen in patients with advanced disease.

The diagnosis of LC can be difficult because the symptoms are often non-specific and can be similar to other neurological conditions. Some of the common symptoms of LC include headache, nausea, vomiting, confusion, memory loss, seizures, and changes in mood or behavior [4]. In some cases, LC may also cause paralysis or weakness in the limbs. Imaging studies, including computed tomography (CT) and magnetic resonance imaging (MRI) of the central nervous system (CNS), may not reveal any significant findings [5]. The presence of malignant cells in the cerebrospinal fluid clinches the diagnosis, although the CSF may be negative at times [6].

Treatment for LC is challenging and often involves a combination of chemotherapy, radiation therapy, and other supportive measures to manage symptoms. Chemotherapy is the primary treatment for LC, and the type of chemotherapy used will depend on the type of cancer and its responsiveness to different drugs. Radiation therapy can also be used to shrink tumors and relieve symptoms. In some cases, surgery may be necessary to remove tumors or relieve pressure on the spinal cord [7].

In addition to chemotherapy and radiation therapy, other supportive measures such as pain management, physical therapy, and rehabilitation can be used to manage symptoms and improve the quality of life for patients with LC.

Despite advances in the treatment of LC, the prognosis for patients with this condition is often poor. The average survival time for patients with LC is between three and six months, although some patients may survive for longer periods of time with aggressive treatment [8]. Palliative care forms the cornerstone of management once the patient is diagnosed with LC, and early palliative care offers better symptom control and patient satisfaction.

## Case presentation

A 52-year-old post-menopausal woman presented to the emergency department in February 2023 with symptoms of severe headache and confusion. The patient had no other positive signs or symptoms. Since she was up and about more than 50% of her waking time and able to carry out any activity of self-care, her performance status was deemed as 2 according to the Eastern Cooperative Oncology Group (ECOG) performance scale [9].

This patient initially presented with a lump in the upper outer quadrant of the right breast a year ago. The patient had no other co-morbidities or symptoms at the time. An ultrasound-guided biopsy of the breast lesion confirmed malignancy, and subsequent immunohistochemistry testing demonstrated negative expression of estrogen (Er), progesterone (Pr), and human epidermal growth factor 2 (HER2/neu) receptors. She underwent a complete work-up, including a bone scan and CT of the whole body, which reported that the tumour was limited to the right breast and axilla. She was staged as a stage 3 tumour according to the American Joint Committee on Cancer (AJCC 8th ed.) [10]. She was initiated on neo-adjuvant systemic therapy with four cycles of doxorubicin, cyclophosphamide, and pembrolizumab, followed by four cycles of paclitaxel, carboplatin, and pembrolizumab. She underwent a partial mastectomy with complete axillary lymph node dissection, and the pathology report found no residual disease.

**Figure 1:**
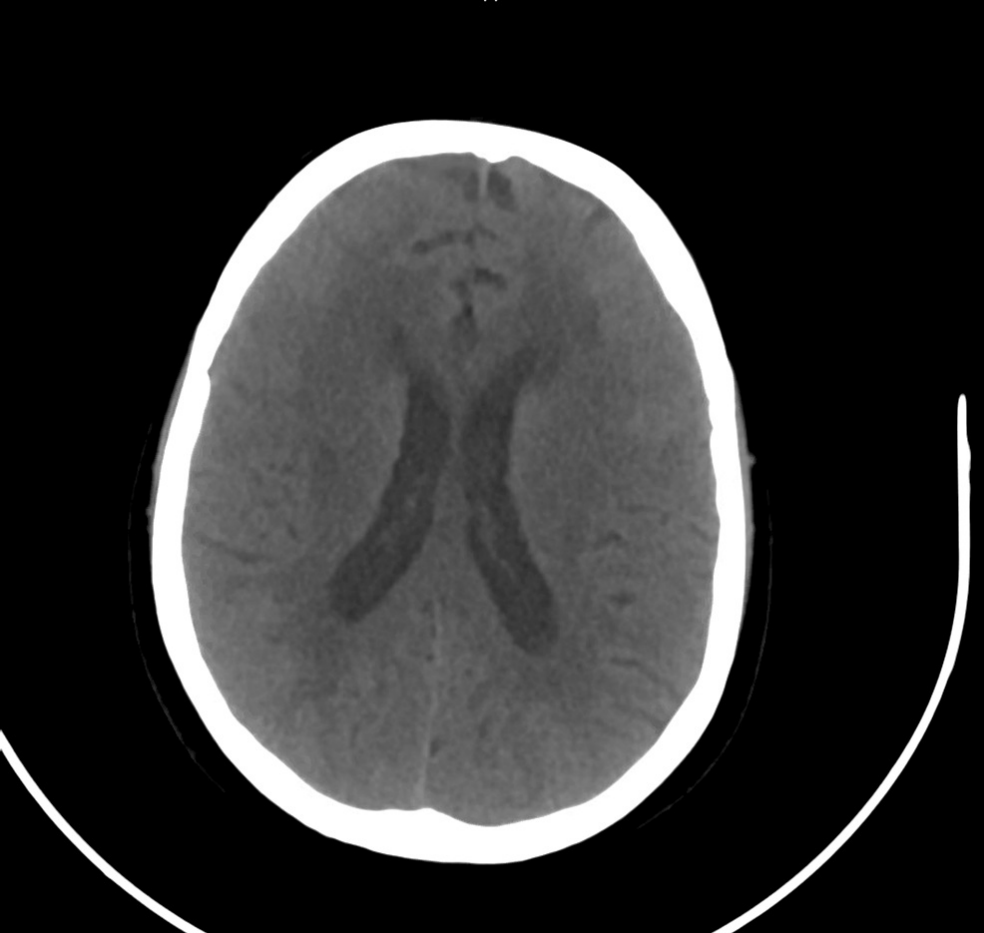
CT brain showing no definite mass lesions.

**Figure 2:**
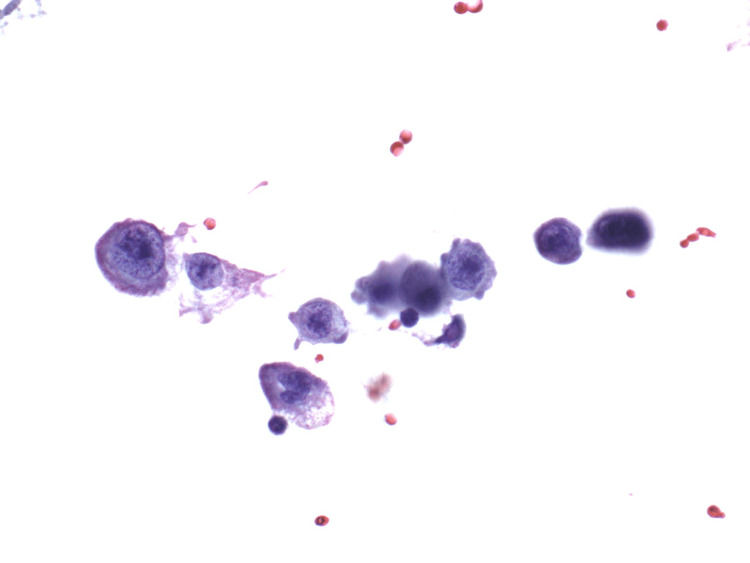
Papanicolaou stain of CSF fluid demonstrates large cells (compare the size to surrounding RBCs) with increased nuclear cytoplasmic ratio and clumped nuclear chromatin. CSF: cerebrospinal fluid.

**Figure 3:**
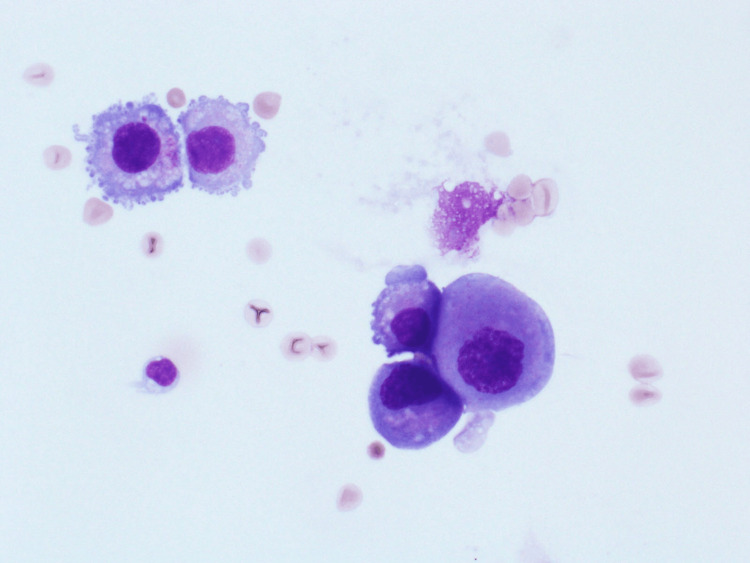
Diff quick stain of CSF shows malignant cells with intense cytoplasm and hyperchromatic nuclei, some of the cells show cytoplasmic blebs. CSF: cerebrospinal fluid.

Six weeks after the surgery, the patient developed a severe headache, nausea, and vomiting. Multiple MRI of the brain and spine revealed no specific abnormality, and a CSF analysis and microscopy reported the presence of malignant cells, confirming leptomeningeal carcinomatosis. The patient underwent intrathecal methotrexate therapy and completed 12 weekly cycles before returning to the hospital emergency department with severe headache and confusion in February 2023. A repeat CT scan of her brain demonstrated no definitive mass lesions (Figure 1), while a repeat craniospinal fluid (CSF) analysis and microscopy revealed malignant cells in the fluid (Figures 2-3), confirming the persistence of leptomeningeal carcinomatosis.

**Figure 4:**
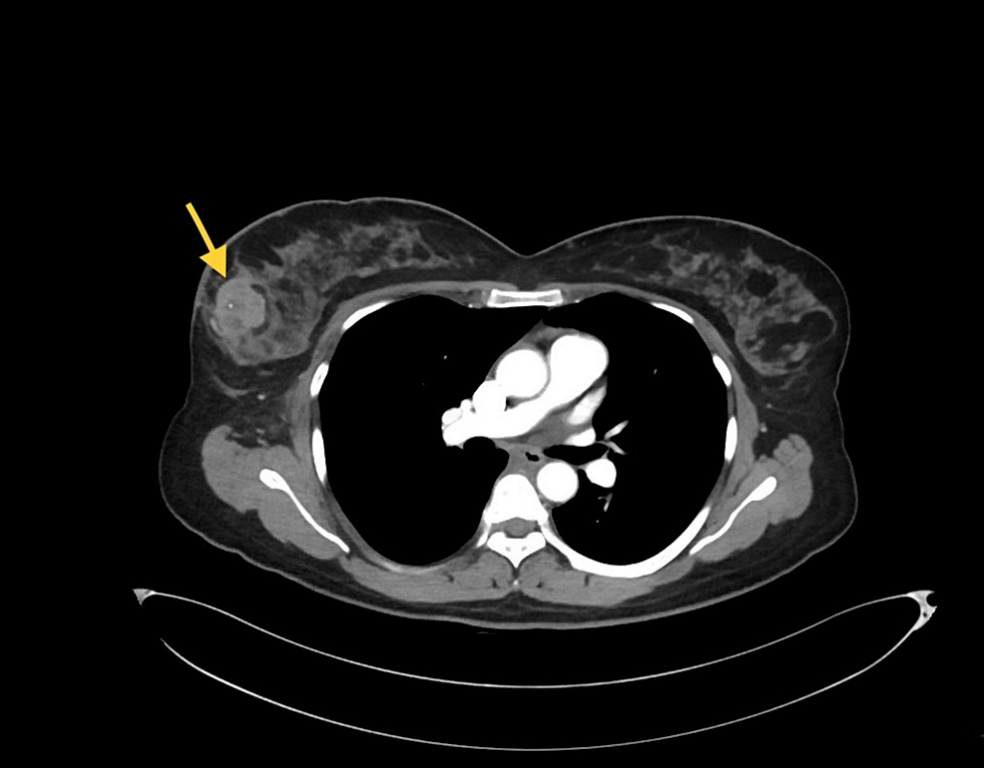
CT chest with intravenous contrast showing the lump in the right breast.

**Figure 5:**
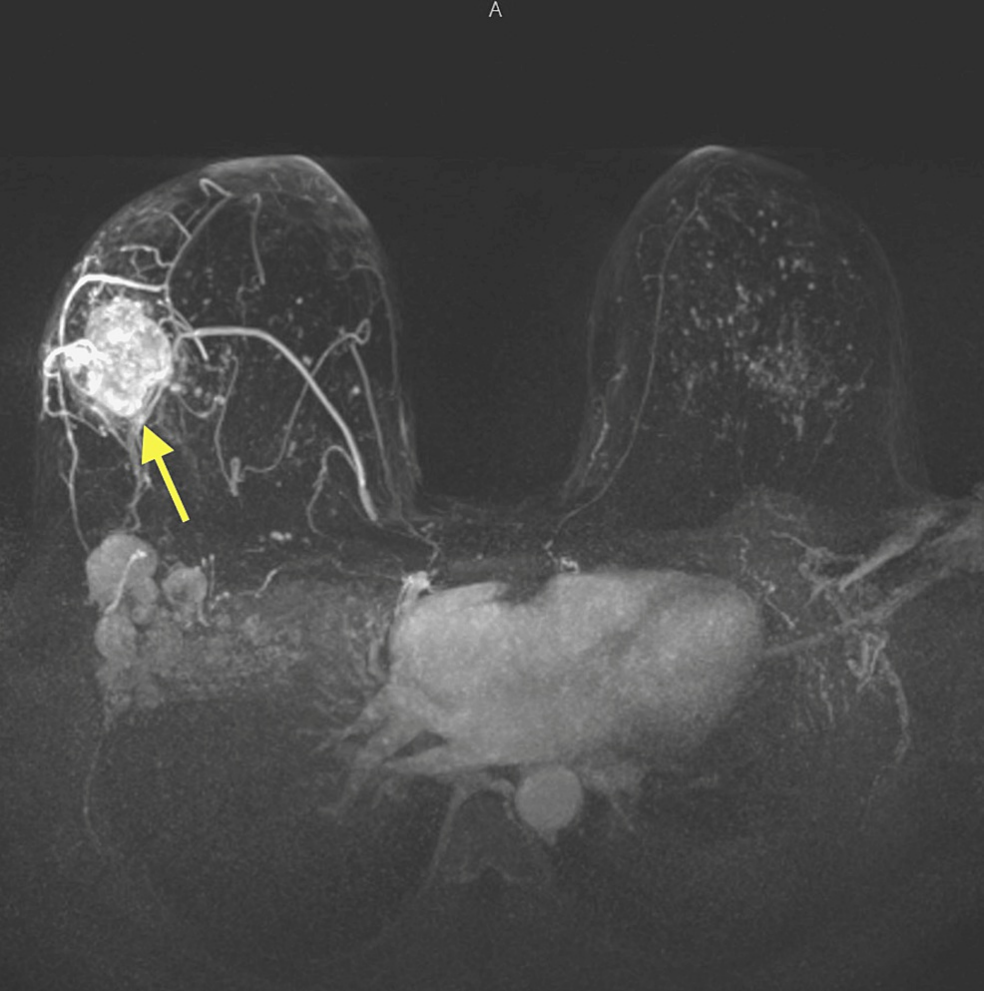
MRI right breast showing the breast mass.

This was deemed to be a progression of her pre-existing triple-negative breast cancer, which was initially diagnosed in January 2022. As mentioned earlier, the patient's triple-negative breast cancer was first identified as an 8 cm × 5 cm lump in her right breast. Imaging studies, including a CT of the chest (Figure 4) and an MRI of the breast (Figure 5), confirmed the presence of the mass and ipsilateral axillary lymph nodes.

Since the patient was getting worse and the leptomeningeal carcinomatosis was persistent, the medical oncology team referred the patient to palliative care. The palliative care team initiated medical management to control the patient's symptoms, which included the use of opioid analgesics, corticosteroids, anti-emetics, and laxatives. Dexamethasone 4 mg intravenously, three times a day, along with pantoprazole 40 mg intravenously, were initiated. Codiene-paracetamol controlled the pain well, and polyethylene glycol was used to prevent any constipation caused by opioids. Ondansetron 8 mg intravenously once a day and Metoclopramide 10 mg intravenously as pro re nata (PRN) were initiated to control nausea and vomiting. The patient and her family were provided with detailed information about the disease, the prognosis, and the importance of an advanced care plan. The palliative care team ensured that the patient's preferences were the top priority and committed to achieving complete symptom control and optimal quality of life for the patient and her family.

Within 24 hours of meeting with the palliative care team, the patient's symptoms were controlled, and the patient reported minimal pain (2/10) according to the numerical pain scale [11]. The patient and her husband expressed a desire to return to their home country for further management and to be closer to their family. The patient was discharged and travelled to her home country without any complications or symptoms.

## Discussion

The World Health Organisation defines palliative care as "an approach that improves the quality of life of patients and their families facing the problems associated with life-threatening illness through the prevention and relief of suffering by means of early identification and impeccable assessment and treatment of pain and other problems, physical, psychosocial, and spiritual" [12].

Every year, approximately 56.8 million individuals require palliative care, and only 14% of those who need this type of care around the world actually receive it [13]. The World Health Organization recognizes the importance of early intervention for patients with a terminal illness, as outlined in their definition of palliative care. However, many patients with advanced cancer receive aggressive curative treatments that are known to be futile, resulting in a loss of quality of life for the patient and negatively impacting their family and caregivers [14]. Studies have shown that patients who continue active therapies such as aggressive systemic therapy are more likely to die during treatment and experience negative psychological and psychiatric repercussions for their caregivers [15].

Early recognition of the need for palliative care and appropriate referral can help build a rapport between clinicians, patients, and their families, leading to better symptom management and ultimately better end-of-life decision-making [16,17]. Early palliative care also reduces the needless use of limited healthcare resources and debilitating treatments, such as continuing chemotherapy cycles in advanced cancers and hospital interventions at the end of life [18].

International recommendations advise early palliative care as it enhances the quality of life through better symptom management, including reducing depression and improving survival. Patients receiving early palliative care have reported greater satisfaction with care [19]. Therefore, early palliative care must be initiated when advanced cancer is diagnosed and should include a complete care assessment, patient-centered treatment, symptom management, knowledge enhancement regarding the disease and prognosis, and psychologic coping [20].

Several barriers have been identified that hinder appropriate palliative and end-of-life care for patients, with delayed referral to a palliative care specialist team being one of the most critical barriers. Late referral often results in difficulties in achieving the goal of improving the patient's quality of life. While evidence supports the benefits of early palliative care in patients with advanced cancers, the question of barriers to early referral and proper utilization remains [21].

One of the significant obstacles is the lack of understanding of the term "palliative care" and confusion with terminal care, which hinders early referral [22]. The lack of a standardized tool to predict mortality in patients with advanced cancers also presents a challenge, as clinicians struggle with disease prognostication and the timing for referral. In addition, physicians' and patients' overly optimistic outlooks often contribute to inaccuracy in mortality prediction [23].

To overcome these barriers, it is crucial to define early palliative care more precisely and present it to patients and healthcare professionals [22]. Standardizing tools to predict mortality in patients with advanced cancers, as well as providing education and training to physicians, can also help improve early referral rates [23].

## Conclusions

In conclusion, LC is a rare but serious complication of cancer that can cause a variety of symptoms. The diagnosis of LC can be difficult due to the non-specific symptoms, but a combination of imaging studies and a lumbar puncture can provide a definitive diagnosis. Treatment for LC is challenging and often involves a combination of chemotherapy, radiation therapy, and other supportive measures. Despite advances in treatment, the prognosis for patients with LC is often poor, with an average survival time of 3 to 6 months.

This case report demonstrates effectively the importance of early referral to a specialized palliative care unit, which resulted in a better quality of life for the patient and the family.
